# Supplementary material for: Crystal structure of SARS-CoV-2 Orf9b in complex with human TOM70 suggests unusual virus-host interactions
Source: Nat Commun. 2021 May 14;12:2843. doi: 10.1038/s41467-021-23118-8 (PMC8121815; doi:10.1038/s41467-021-23118-8)
Supplement: Supplementary file 1 — Supplementary information [file 41467_2021_23118_MOESM1_ESM.pdf]

Supplementary Information for

Crystal Structure of SARS-CoV-2 Orf9b in Complex with Human TOM70 Suggests Unusual Virus-Host Interactions

Xiaopan Gao[#,1,2], Kaixiang Zhu[#,1], Bo Qin[#,1,2] , Vincent Olieric[3], Meitian Wang[3] and Sheng Cui[*,1,2]

[1]NHC Key Laboratory of Systems Biology of Pathogens, Institute of Pathogen Biology, Chinese Academy of Medical Sciences and Peking Union Medical College, Dong Dan San Tiao No.9, Beijing, 100730, P. R. China.

[2]Sanming Project of Medicine in Shenzhen, National Clinical Research Center for Infectious Diseases, Shenzhen Third People's Hospital, Southern University of Science and Technology

[3]Swiss Light Source Paul Scherrer Institut. Villigen PSI 5232, Switzerland

*Correspondence should be addressed to: S.C. (cui.sheng@ipb.pumc.edu.cn)

#These authors contributed equally to this work.

Supplementary Figures

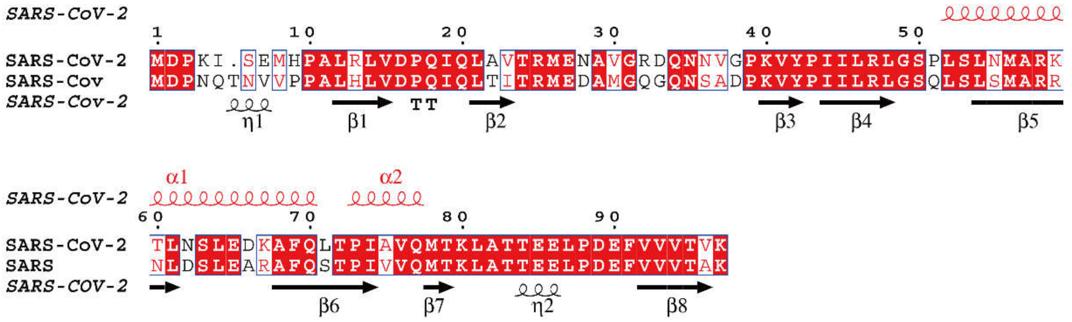

Supplementary Figure 1. Structure-based multiple sequence alignment of orf9b from SARS-CoV and SARS-CoV-2

The sequence alignment of orf9b from SARS-CoV-2 and SARS-CoV. Secondary structure elements of SARS-CoV-2 orf9b bound by human TOM70 are shown at the top. Secondary structure elements of SARS-CoV-2 orf9b in dimer are shown at the bottom. Invariant residues are shown with red background and conserved residues are shown in red fonts. The sequence alignment was calculated and rendered using the software Clustal Omega and ESPript 3.0.

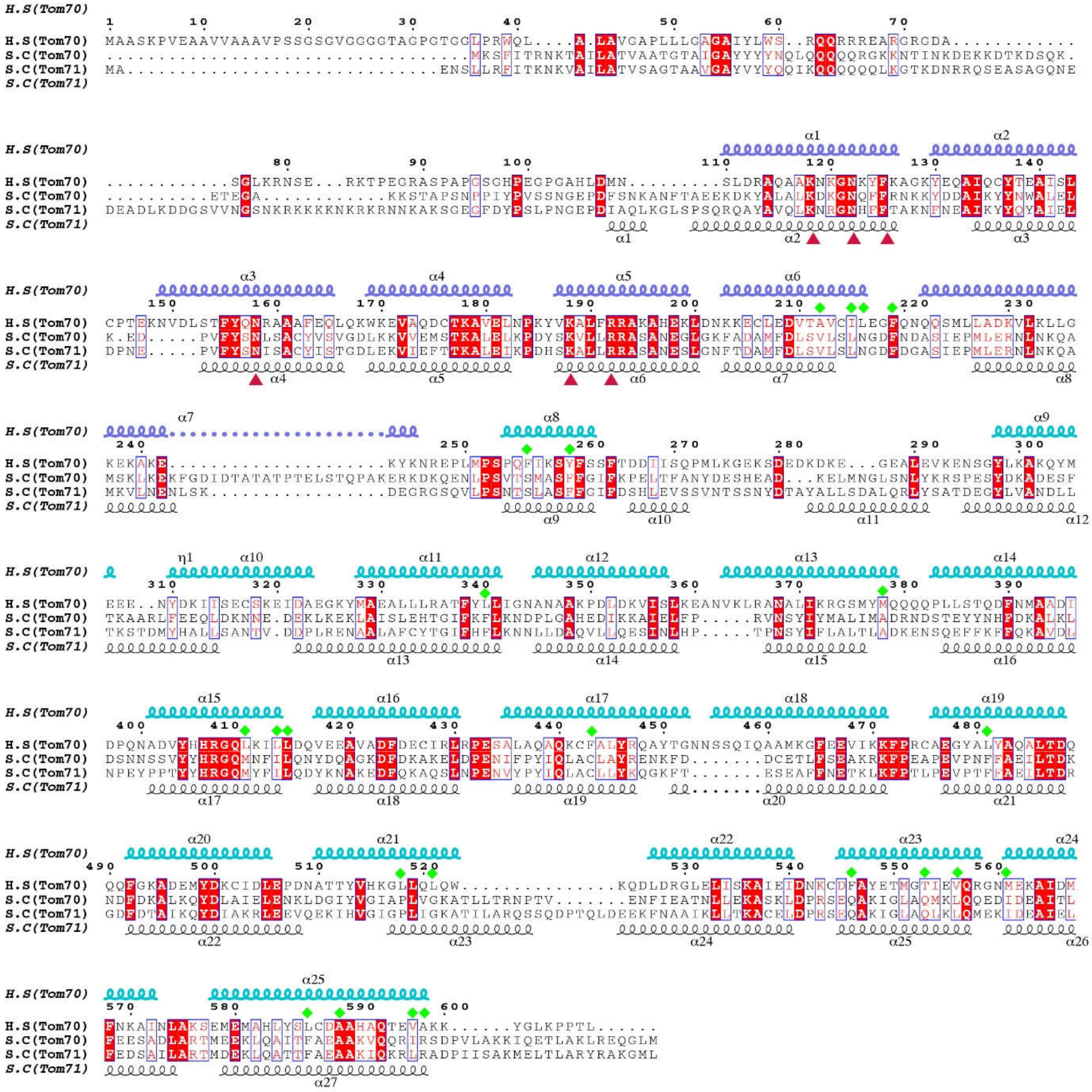

Supplementary Figure 2. Structure-based multiple sequence alignment of Tom70 and Tom71 family members

The multiple sequence alignment of TOM70 and TOM71 proteins from *H.sapiens(H.s)* and *S.cerevisiae (S.c)*.Secondary structure elements of human TOM70 are shown at the top; NTD helices are colored in blue, CTD helices are colored in cyan; residues involving in non-polar contacts with SARS-CoV-2 orf9b are indicated with green diamonds. Secondary structure elements of yeast TOM71 are shown at the bottom; resdiues involving in recognition of the EEVD motif are indicated with red triangles. Invariant residues are shown with red background and conserved residues are shown in red fonts. The sequence alignment was calculated and rendered using the software Clustal Omega and ESPript 3.0.

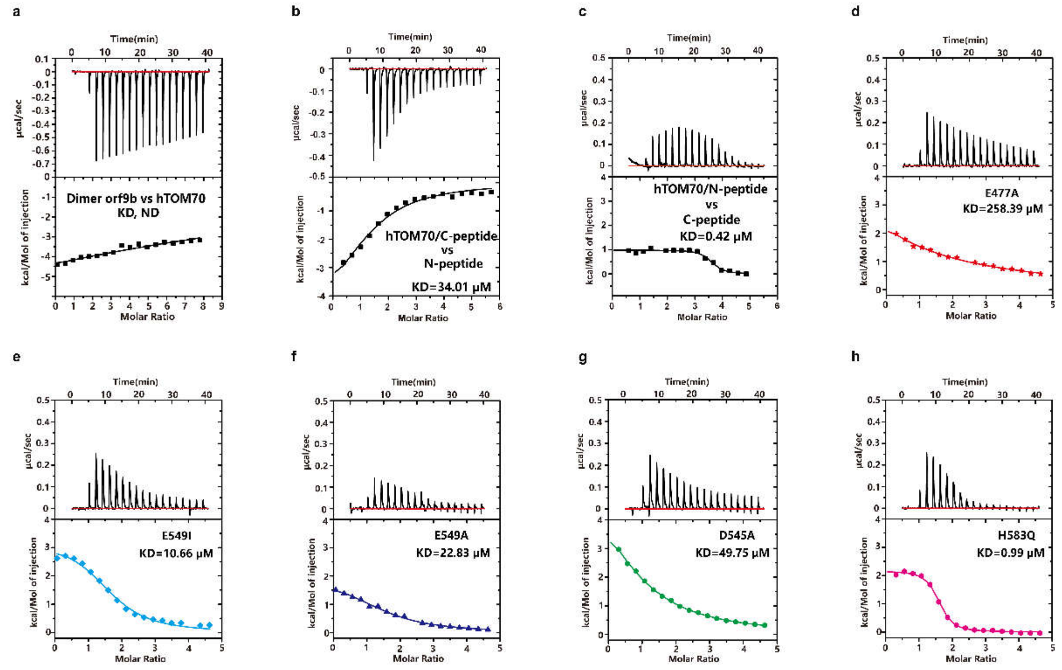

Supplementary Figure 3. Thermodynamics of the binding between SARS-CoV-2 orf9b and hTOM70 variants.

a) Binding isotherm for the interaction between hTOM70 and homodimeric SARS-CoV-2 orf9b.
b) Binding isotherm for the interaction between the hTOM70/C-peptide complex (pre-incubated with molar ratio 1:3) and the N-peptide.
c) Binding isotherm for the interaction between the hTOM70/N-peptide complex (pre-incubated with molar ratio 1:3) and the C-peptide.

d-h. Binding isotherms for the interactions between hTOM70 mutants (E477A red, E549I light blue, E549A dark blue, D545A green and H538Q magenta) and the C-peptide derived from SARS-CoV-2 orf9b. The calculated KD values are indicated.

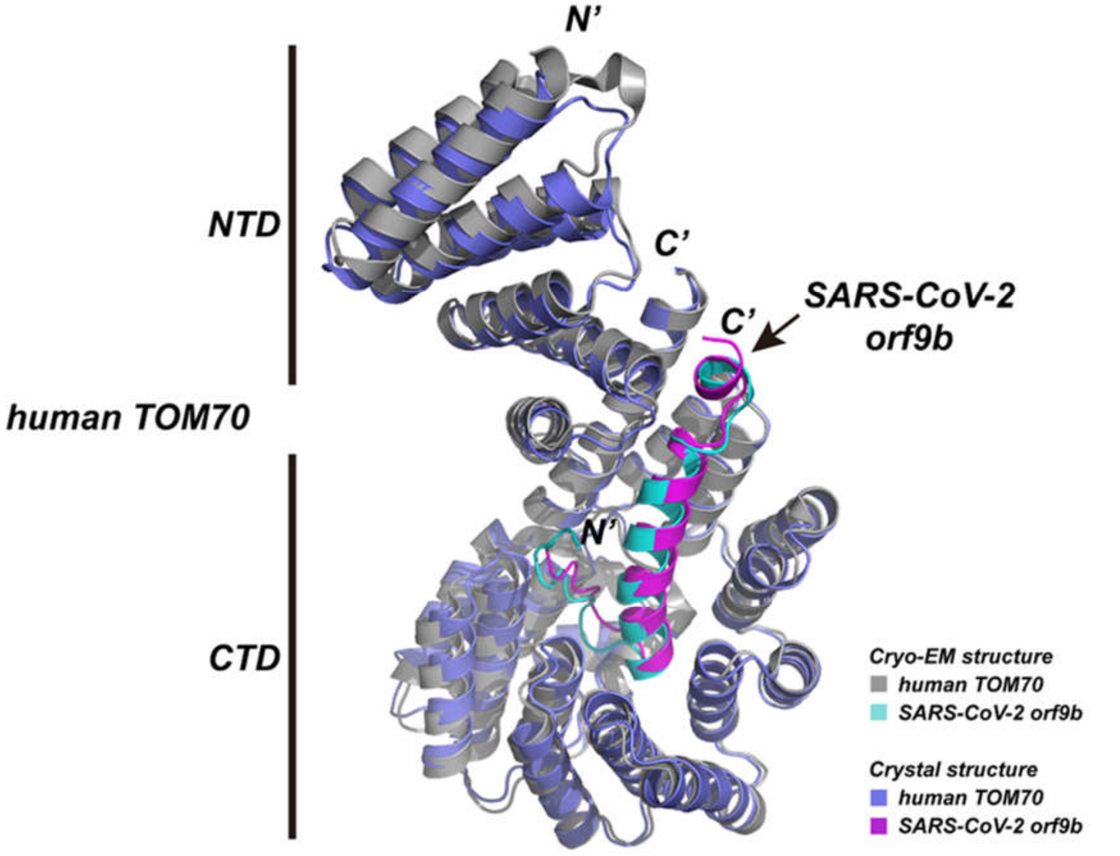

Supplementary Figure 4. Superimposition of the cryo-EM structure of human TOM70/SARS-CoV-2 orf9b complex with the crystal structure of the complex.

The cryo-EM structure of human TOM70/SARS-CoV-2 orf9b complex (PDB ID: 7KDT) is superimposed with the crystal structure of the complex. In the cryo-EM structure, human TOM70 is colored gray and the bound SARS-CoV-2 orf9b is colored cyan. In the crystal structure, human TOM70 is colored blue, and the bound SARS-CoV-2 orf9b is colored magenta.

Supplementary Tables

Supplementary Table 1

Data collection and refinement statistics.

	**SARS-CoV-2 orf9b in complexed Tom70 (PDB ID:7DHG)**
**Data collection**	
Space group	$P2_12_12_1$
Cell dimensions	
a, b, c (Å)	54.66 79.44 124.03
α, β, γ ( )	90.00, 90.00, 90.00
X ray source	
Wavelength (Å)	0.98
Data range (Å)	66.89-2.20(2.33-2.20)
Reflections unique	51,880[a]
R_{sym} [b] (highest resolution shell)	0.099 (1.355)
$I / \sigma I$ (highest resolution shell)	10.33 (0.71)
Completeness (%) (highest resolution shell)	98.1 (88.9)
Redundancy (highest resolution shell)	5.78 (2.99)
**Refinement**	
Resolution range (Å)	50.01-2.20
Reflections, cut-off, % reflections in cross validation	51,681[a], 1.33, 4.94
R_{work} [c]/ R_{free} [d] (highest resolution shell)	0.2241/0.2668 (0.3455/ 0.3708)
**Atoms**	
Non-hydrogen protein atoms	4018
Protein	4018
Solvent	61
B-factors average (Å^2)	58.99
Protein (Å^2)	59.03
Ligands (Å^2)	0
Solvent (Å^2)	56.75
**r.m.s.d**	
Bond lengths (Å)	0.002
Bond angles ( )	0.444
% residues in favored regions, allowed regions, outliers in Ramachandran plot	98,2,0

Values in parentheses are for the highest-resolution shell.

[a] Friedel pairs are treated as different reflections

[b] $R_{sym} = \sum_{hkl}\sum_j |I_{hkl,j} - I_{hkl}|/\sum_{hkl}\sum_j I_{hkl,j}$, where I_{hkl} is the average of symmetry-related observations

of a unique reflection
[c] $R_{work} = \sum_{hkl} ||F_{obs}(hkl)|-|F_{calc}(hkl)||/\sum_{hkl}|F_{obs}(hkl)|$.
[d] R_{free}= the cross-validation R factor for 5% of reflections against which the model was not refined.

Supplementary Table 2

Thermodynamic parameters of the binding between hTOM70/SARS-CoV-2 orf2a and synthetic peptides.

Binding partners	N	K_a, M^{-1}	K_d , μM	ΔH, cal/mole	ΔS, cal/mole/deg
**hTOM70 vs C-peptide**	2.2	$1.04*10^6±3.11*10^5$	0.96	2,651±65	36.4
**hTOM70/SARS-CoV-2 orf9b vs C-peptide**	ND	ND	ND	ND	ND
**hTOM70/N-peptide vs C-peptide**	3.48	$2.39*10^6±9.57*10^5$	0.42	981±24	32.5
**hTOM70 vs N-peptide**	1.5	$3.91*10^5±3.21*10^4$	2.56	- 4,150±50	11.7
**hTOM70/SARS-CoV-2 orf9b vs N-peptide**	0	$1.37*10^4±4.97*10^3$	72.99	$-1.38*10^6±2.2*10^8$	-4,600

**hTOM70/C-peptide vs N-peptide**	1.3	$2.94*10^4±6.91*10^3$	34.01	- 5,387±964	2.37
**hTOM70 vs dimeric SARS-CoV-2 orf9b**	ND	ND	ND	ND	ND
**hTOM70 E477A vs C-peptide**	0	$3.87*10^3±1.56*10^3$	258.39	$3.67*10^5*±1.57*10^7$	1,250
**hTOM70 E549I vs C-peptide**	1.7	$9.38*10^4±1.54*10^4$	10.66	3,234±129	33.6
**TOM70 E549A vs C-peptide**	1.5	$4.38*10^4±6.29*10^3$	22.83	2,033±120	28.1
**hTOM70 D545A vs C-peptide**	1.0	$2.01*10^4±1.28*10^3$	49.75	7,438±582	44.6
**hTOM70 H583Q vs C-peptide**	1.5	$1.01*10^6±1.34*10^5$	0.99	2,170±28	34.7

N, stoichiometry

Ka, affinity constant; standard deviation did not exceed ±10%.

KD, dissociation constant; calculated as 1/Ka.

ΔH, enthalpy variation; standard deviation did not exceed ±10%.

ΔS, entropy variation.

ND, not determined

Supplementary Table 3

Optimized genes of Orf9b and TOM70 in this study

Gene names	Optimized genes sequence
Orf9b	>ATGGACCCGAAGATCAGCGAGATGCACCCGGCGCTGCGTCTGGTTGATCCG<br>CAGATTCAACTGGCGGTTACCCGTATGGAAAACGCGGTGGGTCGTGACCAGA<br>ACAACGTTGGCCCGAAAGTGTACCCGATCATTCTGCGTCTGGGTAGCCCGCT<br>GAGCCTGAACATGGCGCGTAAGACCCTGAACAGCCTGGAGGACAAAGCGTT<br>CCAGCTGACCCCGATCGCGGTTCAAATGACCAAGCTGGCGACCACCGAGGA<br>ACTGCCGGATGAATTTGTGGTTGTGACCGTGAAATAA
TOM70	>ATGGCGGCGAGCAAACCGGTTGAAGCGGCGGTTGTTGCGGCAGCGGTTCC<br>GTCTAGCGGTAGCGGTGTTGGTGGCGGTGGCACCGCGGGTCCGGGCACCGG<br>TGGCCTGCCGCGTTGGCAGCTGGCGCTGGCTGTGGGCGCCCCGCTGCTGCTG<br>GGTGCGGGCGCGATCTACCTGTGGAGCCGTCAGCAGCGTCGTCGTGAAGCA<br>CGTGGTCGTGGCGACGCGTCGGGTCTGAAACGTAACTCTGAACGTAAAACC<br>CCGGAAGGCCGTGCATCTCCGGCGCCGGGTAGCGGCCACCCGGAAGGTCCG<br>GGTGCACACCTGGATATGAACAGCCTGGATCGCGCGCAGGCGGCGAAAAATA<br>AAGGTAACAAATACTTCAAAGCGGGTAAATACGAACAGGCGATCCAGTGCTA<br>TACTGAAGCAATCTCCCTGTGTCCGACCGAGAAAAACGTTGATCTGAGCACT<br>TTCTACCAGAACCGTGCCGCGGCCTTCGAACAGCTGCAGAAATGGAAAGAA<br>GTGGCTCAGGATTGTACTAAAGCTGTTGAACTGAACCCGAAATACGTGAAAG<br>CACTGTTCCGTCGTGCGAAAGCGCACGAAAAACTGGATAACAAAAAAGAAT<br>GCCTGGAGGACGTTACCGCTGTTTGCATCCTGGAAGGTTTCCAGAACCAGCA<br>GAGCATGCTGCTGGCAGACAAAGTCCTGAAACTGCTGGGTAAAGAAAAAGC<br>TAAAGAAAAATATAAAAACCGCGAACCGCTGATGCCGTCCCCGCAGTTCATT<br>AAATCCTACTTCAGCTCCTTCACCGATGATATCATTTCTCAGCCGATGCTGAA<br>AGGTGAAAAAAGCGATGAAGATAAAGATAAAGAAGGTGAAGCTCTGGAAGT<br>TAAAGAAAACTCCGGTTATCTGAAAGCAAAACAGTATATGGAAGAAGAAAA<br>CTATGATAAAATTATTAGCGAATGCTCTAAAGAAATTGATGCAGAAGGTAAAT<br>ACATGGCGGAAGCCCTGCTGCTGCGCGCGACCTTTTACCTGCTGATCGGCAA<br>CGCGAACGCGGCTAAACCGGATCTGGATAAAGTTATCAGCCTGAAAGAAGCA<br>AACGTTAAACTGCGTGCTAACGCGCTGATTAAACGTGGCTCCATGTATATGCA<br>GCAGCAGCAGCCGCTGCTGTCTACTCAGGATTTCAATATGGCTGCTGACATTG<br>ATCCGCAGAACGCTGATGTTTACCACCACCGCGGCCAGCTGAAAATCCTGCT

	GGATCAGGTTGAAGAAGCTGTTGCAGATTTCGATGAATGTATCCGTCTGCGTCCGGAATCTGCGCTGGCGCAGGCGCAGAAATGCTTCGCTCTGTACCGTCAGGCATACACCGGCAACAACAGCAGCCAGATTCAGGCTGCGATGAAAGGCTTCGAAGAAGTGATCAAAAAATTTCCGCGTTGTGCGGAAGGTTACGCACTGTACGCGCAGGCGCTGACCGATCAGCAGCAGTTCGGTAAAGCCGATGAAATGTATGATAAATGCATCGATCTGGAACCGGACAACGCTACTACCTACGTTCATAAAGGCCTGCTGCAGCTGCAATGGAAACAGGATCTGGATCGTGGTCTGGAACTGATTTCTAAAGCTATCGAAATCGATAACAAATGTGACTTCGCTTACGAAACCATGGGTACCATCGAAGTTCAGCGTGGCAACATGGAAAAAGCCATCGATATGTTCAACAAAGCGATCAACCTGGCGAAATCTGAAATGGAAATGGCTCACCTGTACAGCCTGTGTGATGCGGCTCACGCTCAGACCGAAGTTGCTAAAAAATACGGCCTGAAACCGCCGACCCTG

Supplementary Table 4

List of primers used in this study

Name	Forward primer(5’-3’)	Reverse primer(5’-3’)
pETDuet-1-N-6his-Orf9b (MCS-1）	CATCACCACAGCCAGGATATGGACCCGAAGATCAGC	TTATGCGGCCGCAAGCTTTTATTTCACGGTCACAACCACA
pETDuet-1-TOM70(60-608)（MCS-2）	GAAGGAGATATACATATGAGCCGTCAGCAGCGTCGT	TTCTTTACCAGACTCGAGTTACAGGGTCGGCGGTTT
pETDuet-1-TOM70(106-608)（MCS-2）	GAAGGAGATATACATATGCTGGATATGAACAGCCTGGAT	TTCTTTACCAGACTCGAGTTACAGGGTCGGCGGTTT
pETDuet-1-TOM70(235-608)（MCS-2）	GAAGGAGATATACATATGCTGGGTAAAGAAAAAGCTAAAG	TTCTTTACCAGACTCGAGTTACAGGGTCGGCGGTTT
pET28a-TOM70(106-608)-C-his	GAAGGAGATATACCATGCTGGGTAAAGAAAAAGCTAAAG	GTGGTGGTGGTGGTGGTGCAGGGTCGGCGGTTTCA
pET28a-N-his-Sumo-Orf9b	CAGATTGGTGGATCCATGGACCCGAAGATCAGC	TGGTGGTGGTGCTCGAGTTATTTCACGGTCACAACCACA
pET28a-TOM70(106-608)-C-his(E549A)	GACTTCGCTTACGCAACCATGGGTACC	GGTACCCATGGTTGCGTAAGCGAAGTC
pET28a-TOM70(106-608)-C-his(E549I)	CAAATGTGACTTCGCTTACATAACCATGGGTACCATCGAAG	CTTCGATGGTACCCATGGTTATGTAAGCGAAGTCACATTTG
pET28a-TOM70(106-608)-C-his(H583Q)	GAAATGGAAATGGCTCAACTGTACAGCCTGTGTG	CACACAGGCTGTACAGTTGAGCCATTTCCATTTC
pET28a-TOM70(106-608)-C-his(E477A)	GCGTTGTGCGGCAGGTTACGCACTGTA	TACAGTGCGTAACCTGCCGCACAACGC
pET28a-TOM70(106-608)-C-his(D545A)	CGATAACAAATGTGCCTTCGCTTACGAAACC	GGTTTCGTAAGCGAAGGCACATTTGTTATCG
